# Supplementary material for: Sequence-Dependent Nanofiber Structures of Phenylalanine and Isoleucine Tripeptides
Source: Int J Mol Sci. 2020 Nov 10;21(22):8431. doi: 10.3390/ijms21228431 (PMC7696242; doi:10.3390/ijms21228431)
Supplement: Supplementary file 1 [file ijms-21-08431-s001.pdf]

Supplementary information

Sequence-Dependent Nanofiber Structures of Phenylalanine and Isoleucine Tripeptides

Qinsi Xiong,[1] *Ziye Liu,*[1] *Wei Han*[1,*]

[1]State Key Laboratory of Chemical Oncogenomics, School of Chemical Biology and Biotechnology, Peking University Shenzhen Graduate School, Shenzhen 518055, China

[*]Corresponding author: Email: hanw@pkusz.edu.cn

Definitions of Conformational states of tripeptides

Conformational space of a tripeptide can be divided into discrete conformational states based on conformations of individual amino acid units in the tripeptide (Figure S1), which was defined by the Ramachandran (ϕ, ψ) plot. Four local minima are located on the Ramachandran (ϕ, ψ) map, namely a right-handed state (α_R), a left-handed α helical state (α_L), a β strand (β) state and a polyproline II state (PPII)[1]. The specified parameters for these regions are listed in **Table S2**. Based on our previous work[2], β and PPII conformation state of the N-terminal amino acid share a very similar ψ range (~90° - ~180°) and are separated by a small barrier. Also, tripeptides in these two conformational states have the same θ_1 angle. Thus, they were merged into one state so as to reduce the total number of states from 64 (4^3) to 48 (3×4^2). Of note, the merged state at the N-terminal residue was still labeled "β".

Conformationally constrained simulations

Additional harmonic potentials were applied in conformationally constrained simulations to keep tripeptides constrained in particular conformational states:

$$E_{\text{cons}} = \sum_N \sum_{R\in\{1,2,3\}} \frac{1}{2}\left(K_{\phi_{R,N}}\left(\phi_{R,N} - \mathring{\phi}_{R,N}\right)^2 + K_{\psi_{R,N}}\left(\psi_{R,N} - \mathring{\psi}_{R,N}\right)^2\right), \text{[S1]}$$

where $K_{\phi_{R,N}}$ and $K_{\psi_{R,N}}$ determine the "stiffness" of the potential, and $\mathring{\phi}_{R,N}$ and $\mathring{\psi}_{R,N}$ are the equilibrium values of backbone dihedral $\phi_{R,N}$ and $\psi_{R,N}$ of the R_{th} residue in the N_{th} monomer. For each conformational state , a set of $\{K_{\phi_R}, \mathring{\phi}_R, K_{\psi_R}, \mathring{\psi}_R | R=1,2,3\}$ parameters were applied to restrain tripeptide conformations. These parameters were derived by fitting the constrained simulation results with PACE to the corresponding regions in the ϕ and ψ map of FFF assemblies obtained from the unconstrained self-assembly simulations. The fitting results are shown in **Figure S3** and the resulting parameters of the constraining forces are summarized in **Table S5**.

Table S1. Summary of simulations using PACE force field conducted in this study.

Tripeptide Sequence	Numbers of tripeptides/particles	Constrained Conformational state	Simulation Type (temperature)	Simulation time (us)[a]
FFF	30/3207	No	Simulated annealing and conventional MD (310K-370K)	RUN1, 3.0; RUN2, 2.2; RUN3: 2.5
FFI	30/3145			RUN1, 3.3; RUN2, 2.4; RUN3: 2.5
FIF	30/3122		conventional MD (310K)	RUN1: 2.1; RUN2: 2.9; RUN3:2.5
FII	30/3075			RUN1: 2.5; RUN2: 2.3; RUN3:2.0
IFF	30/3131			RUN1: 2.1; RUN2: 2.4; RUN3:2.2
IFI	30/3068			RUN1: 2.3; RUN2: 2.5; RUN3:2.0
IIF	30/3063		Simulated annealing and conventional MD (310K-370K)	RUN1, 2.4; RUN2, 3.0; RUN3: 2.7
III	30/3015		conventional MD (310K)	RUN1: 2.6; RUN2: 3.0; RUN3:2.0
FFF	30/3207	$\alpha_R\alpha_R\beta$	Simulated annealing and conventional MD (310K-400K)	RUN1, 2.3; RUN2, 2.0
		βββ		RUN1, 2.7; RUN2, 2.2
FFI	30/3145	$\alpha_R\alpha_R\beta$		RUN1, 2.3; RUN2, 2.0
		βββ		RUN1, 2.3; RUN2, 2.0
FIF	30/3122	$\alpha_R\alpha_R\beta$		RUN1, 2.2; RUN2, 2.2
		βββ		RUN1, 2.0; RUN2, 1.8
FII	30/3075	$\alpha_R\alpha_R\beta$		RUN1, 2.4; RUN2, 2.3
		βββ		RUN1, 1.9; RUN2, 1.8
IFF	30/3131	$\alpha_R\alpha_R\beta$		RUN1, 2.0; RUN2, 2.2
		βββ		RUN1, 2.0; RUN2,1.7
IFI	30/3068	$\alpha_R\alpha_R\beta$		RUN1, 2.4; RUN2, 2.7
		βββ		RUN1, 2.1; RUN2, 1.9
IIF	30/3063	$\alpha_R\alpha_R\beta$		RUN1, 2.1; RUN2, 2.0
		βββ		RUN1, 3.2; RUN2, 3.2
III	30/3015	$\alpha_R\alpha_R\beta$		RUN1, 2.8; RUN2, 2.5
		βββ		RUN1, 2.1; RUN2, 1.8

(a) Due to the stochastic nature of self-assembly processes, the time needed for observing the assembly of ordered structures varied significantly in different simulations. To save computation resource, we stopped the simulations if ordered assembled structures formed and were maintained for a period of time (~300ns). As such, there is a variation of simulation time for different runs of the same type of simulations. Also, in the conventional MD simulations of FFF, FFI, IIF and those with conformational constraints, the final assembly structures obtained from independent simulations were difficult to converge, indicating the kinetic traps present in these cases. Therefore, we conducted annealing simulations to prevent systems from being trapped. The temperature range of the annealed simulations was set to be 310-370K for non-constrained simulations and 310-400K for constrained simulations.

Table S2. The (ϕ, ψ) regions in Ramachandran map used to define two typical conformational states of an amino acid

Conformation	ϕ	ψ
α_R	(-180°, 0°)	(-120°, 30°)
	(-180°, -100°)	(30°, 60°)
β	(-180°, -100°)	(90°, 180°)
	(150°, 180°)	(90°, 180°)
	(-180°, -100°)	(-180°, -170°)
PPII	(-100°, 0°)	(90°, 180°)
α_L	(0°, 180°)	(-30°, 80°)

Table S3. Summary of probability of conformational states in solution[a]

**FFF**		**FFI**		**FIF**		**FII**	
$\beta\alpha_R\alpha_R$	0.24829468	$\beta\alpha_R\alpha_R$	0.17449849	$\beta\alpha_R\alpha_R$	0.13711648	$\beta\alpha_R\alpha_R$	0.19616838
$\beta\beta\beta$	0.14624829	$\beta\beta\beta$	0.12613355	$\beta\alpha_R\beta$	0.1099647	$\alpha_R\alpha_R\alpha_R$	0.17781975
$\beta\alpha_R$PPII	0.14270123	$\beta\alpha_R$PPII	0.1082715	$\beta\alpha_R$PPII	0.10887863	$\beta\alpha_R$PPII	0.0849973
$\beta\beta$PPII	0.14160982	$\beta\alpha_R\beta$	0.09563067	βPPIIβ	0.10181917	βPPIIβ	0.08094981
βPPIIPPII	0.09795362	$\beta\beta$PPII	0.09178346	$\beta\beta$PPII	0.09856096	$\beta\alpha_R\beta$	0.07717215
		βPPIIβ	0.08134103	$\beta\beta\beta$	0.09666033	$\beta\beta\beta$	0.06583918
				βPPIIPPII	0.06163454	$\beta\beta$PPII	0.06502968
**IFF**		**IFI**		**IIF**		**III**	
$\beta\beta\beta$	0.25569128	$\beta\alpha_R\alpha_R$	0.23655622	$\alpha_R\alpha_R\alpha_R$	0.2177814	$\beta\alpha_R\alpha_R$	0.22032086
$\beta\alpha_R\beta$	0.09661299	$\beta\alpha_R\beta$	0.19038566	$\beta\alpha_R\alpha_R$	0.19521479	$\beta\alpha_R\beta$	0.18262032
$\alpha_R\alpha_R\alpha_R$	0.08856191	βPPIIβ	0.09424226	$\beta\alpha_R$PPII	0.15796629	$\beta\alpha_R$PPII	0.13235294
$\alpha_R\alpha_R\beta$	0.07745697	$\beta\alpha_R$PPII	0.08881043	$\alpha_R\alpha_R$PPII	0.08156607	βPPIIβ	0.11122995
$\beta\alpha_R$PPII	0.07717934	$\alpha_R\alpha_R\alpha_R$	0.07224335	$\alpha_R\beta$PPII	0.05981512	$\beta\beta\beta$	0.07459893
$\beta\alpha_R\alpha_R$	0.0755136	$\beta\beta\beta$	0.06165128	$\beta\alpha_R\beta$	0.0581838		
βPPIIβ	0.06274292						
$\beta\beta\alpha_R$	0.05219323						

(a) The conformation states with an average probability > 5% are listed here.

Table S4. Summary of probability of conformational states in assemblies[a]

**FFF**		**FFI**		**FIF**		**FII**	
βββ	0.62181889	$\alpha_R\alpha_R\beta$	0.51093481	βββ	0.53198782	$\alpha_R\alpha_R\beta$	0.37436312
ββPPII	0.14195445	$\alpha_R\alpha_R\beta$	0.09222792	ββPPII	0.19182707	$\alpha_R\alpha_R\alpha_R$	0.10047373
βPPIIβ	0.07473799	βββ	0.0828779	βPPIIβ	0.07076193	βββ	0.07250068
$\beta\alpha_L$PPII	0.06957351	$\alpha_R\alpha_L$PPII	0.05468549	$\beta\beta\alpha_R$	0.05409921	$\beta\alpha_R\alpha_R$	0.0637831
**IFF**		**IFI**		**IIF**		III	
$\alpha_R\alpha_R\beta$	0.63888775	$\alpha_R\alpha_R\beta$	0.73816251	$\alpha_R\alpha_R\beta$	0.16655251	βββ	0.38358591
$\alpha_R\alpha_R\beta$	0.1579894	$\alpha_R\alpha_R\beta$	0.12825635	$\alpha_R\alpha_R\beta$	0.14651803	$\alpha_R\alpha_R\beta$	0.23475643
$\alpha_R\alpha_R$PPII	0.09485864			$\beta\alpha_R\alpha_R$	0.09887021	βPPIIβ	0.09786132
				βββ	0.05814445	ββPPII	0.0727873

(a) The conformation states with an average probability > 5% are listed here.

Table S5. Parameters for conformational constraints used to fix FFFs to two major conformational states

	**Conformational states**	
	$\alpha_R\alpha_R\beta$	βββ
K_{ϕ_1} **(kJ/mol/rad²)**	40	40
ϕ_1° **(deg)**	-80	-100
K_{ψ_1} **(kJ/mol/rad²)**	40	40
ψ_1° **(deg)**	-50	130
K_{ϕ_2} **(kJ/mol/rad²)**	40	40
ϕ_2° **(deg)**	-90	-120
K_{ψ_2} **(kJ/mol/rad²)**	30	40
ψ_2° **(deg)**	-35	130
K_{ϕ_3} **(kJ/mol/rad²)**	40	40
ϕ_3° **(deg)**	-150	-127
K_{ψ_3} **(kJ/mol/rad²)**	40	40
ψ_3° **(deg)**	140	137

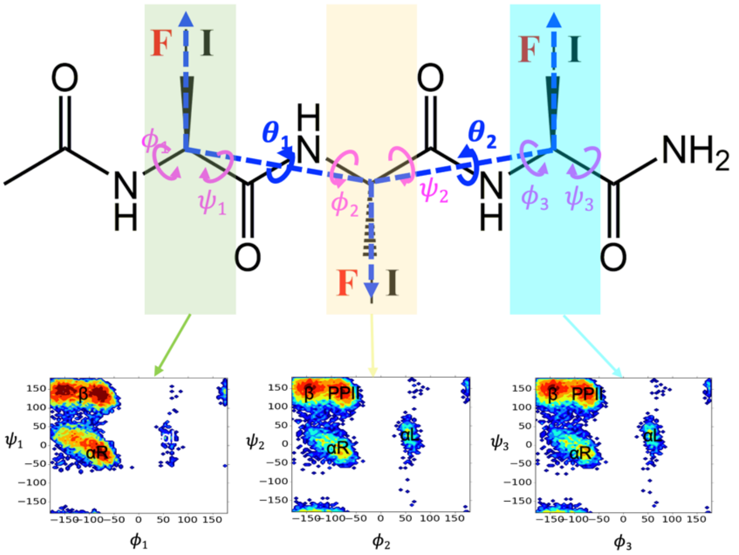

Figure S1. Illustration of conformations of each amino acid of a tripeptide. β-strand substate and polyproline II substate (PPII) of the first amino acid can be further merged into one conformation, still labeled β. The Ramachandran plots were obtained from a simulation of a FFF tripeptide in water using PACE.

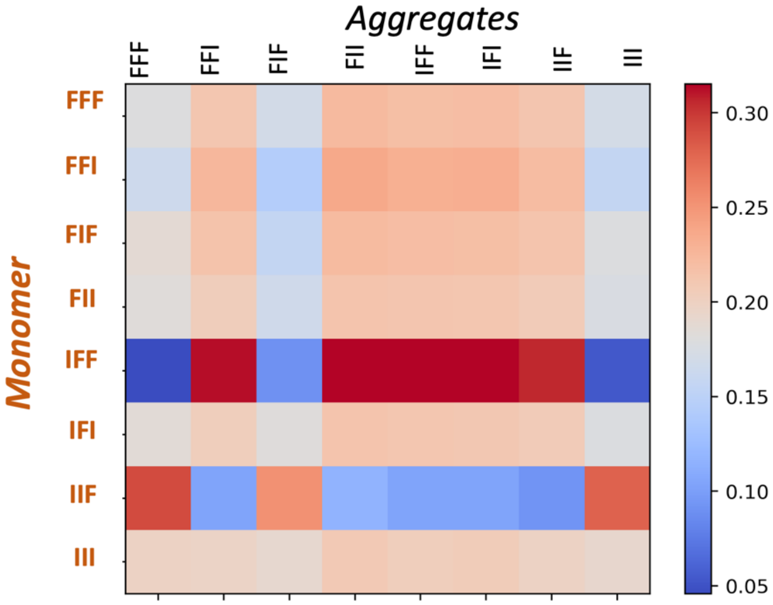

Figure S2. Pairwise root mean square distance (RMSD) between representative conformations in solution states and in assembled states.

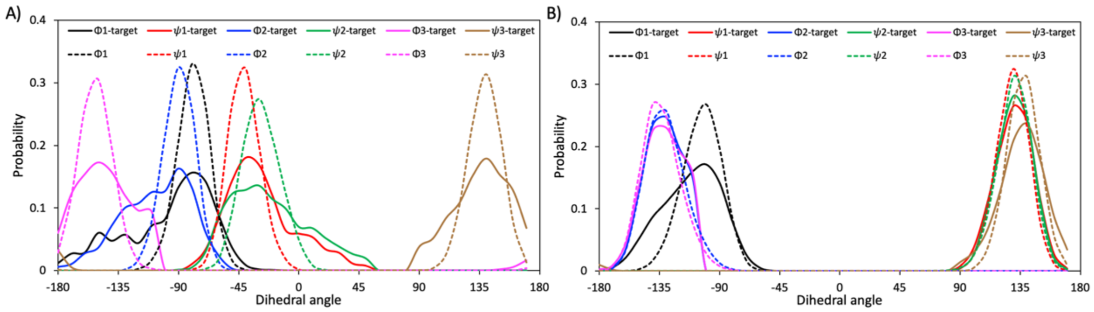

Figure S3. Distributions of backbone dihedral angle ϕ_1 (black), ψ_1 (red), ϕ_2 (blue), ψ_2 (green), ϕ_3 (magenta) and ψ_3 (brown) for $\alpha_R\alpha_R\beta$ (A) and βββ (B) conformational states. The solid curves denote the distributions obtained using the assembled structures of FFFs from unconstrained simulations. Dashed curves were obtained from the simulations in which a FFF was constrained to one of the conformational states using the optimized parameters for the constraining forces.

REFERENCES

1. Pizzanelli, S.; Forte, C.; Monti, S.; Zandomeneghi, G.; Hagarman, A.; Measey, T.J.; Schweitzer-Stenner, R. Conformations of phenylalanine in the tripeptides AFA and GFG probed by combining MD simulations with NMR, FTIR, polarized Raman, and VCD spectroscopy. *J. Phys. Chem. B* **2010**, *114*, 3965–3978.

2. Xiong, Q.; Jiang, Y.; Cai, X.; Yang, F.; Li, Z.; Han, W. Conformation Dependence of Diphenylalanine Self-Assembly Structures and Dynamics: Insights from Hybrid-Resolution Simulations. *ACS Nano* **2019**, *13*, 4455–4468.
